# Notes and Comments

**Published:** 1924-01

**Authors:** 


### NOTES AND COMMENTS.

### STILL ANOTHER MINISTER OF HEALTH.

EVERY General Election brings surprises and disappointments, and, politics apart, general regret will be felt at the approaching departure of Sir W. Joynson-Hicks from the Ministry in which he has for so short a time been seated. His reign has been so brief that it is impossible to say whether he would have been a brilliant success, a signal failure, or a merely respectable incumbent of this great office, and everyone hoped that he would at least enjoy a fair chance of distinguishing himself. As matters stand it is tolerably certain that by the time our next number is published there will be yet another Minister of Health. If that were all we might still be hopeful that the new man would be given "time to turn round" and to evolve something in the nature of a policy which could be steadily pursued. But the position of parties in the House of Commons is such that it is in the highest degree improbable that any Administration which takes office next month can remain long in power. All too soon, therefore, we may expect another General Election and yet another Minister of Health. There have been three Ministers this year, and five since the office was created five years ago; in 1924 there will probably be two more. It is impossible that a great Department of State dealing with the physical welfare of the nation can perform its functions with reasonable satisfaction with an interminable series of temporary chiefs flitting uneasily through its portals. Some measure of stabilisation is becoming imperative. Otherwise we had better put the Ministry into commission and confide it to the experienced hands of the permanent officials whose responsibility of late, in the midst of so many changes, has clearly been greater even than usual.

### PROGRESS OF THE KING'S FUND.

THAT at a time like the present, when the country is taxed more heavily than any other nation in the world, and is at the same time suffering severely from stagnation of trade, King Edward's Hospital Fund for London should be able to distribute the noble sum of £235,000, is a circumstance of real significance. This is £15,000 more than was available a year ago, and in his message to the General Council of the Fund the King was entirely justified in saying that the difficulties facing the voluntary hospital system are being steadily overcome by the methods adopted by it. There have been some windfalls, but the income from subscriptions and donations has, happily, been well maintained. The hospitals are not yet out of the wood, but we may reasonably believe that the voluntary principle has been saved, and the great work now before us is to keep it safe for posterity. The Fund is thoroughly alive to this necessity, and the Prince of Wales, who presided at the meeting of the General Council, announced a very useful development of the activities of the Revenue Committee in enlisting the help of the professions and the exponents of the liberal arts, while attempts are to be made to raise revenue in ways that are not already covered. A very plain caution was given by the Committee on Recovery Branches of London Hospitals about the establishment of such branches. The more we have of them the better, but those who purpose creating them are advised to remember that they are not as cheap as they look, and that they ought to be budgeted for as a form of hospital extension. The committee urge, moreover, that no form of extension should be undertaken without careful consideration both by the hospital and the King's Fund "until the restoration of voluntary hospital finance has been completed." This is obvious commonsense, notwithstanding the urgent need on every hand of undertaking the long overdue work of extension. We must make the best of what we have before we embark upon new enterprises however desirable.

### ORGANISING A HEALTH WEEK.

UPON another page we have set out the reasons which have decided THE HOSPITAL AND HEALTH REVIEW to offer a prize of £100 for the best method of organising a Health Week. We need not repeat those reasons here. Their weight will be recognised by every man and woman who is engaged in the fine work of improving the public health. The number of these men and women—doctors and nurses, and sanitary officials of all grades, to say nothing of the members of Public Health Committees of local governing bodies—is very large. All have had experience in combating disease, in improving the machinery for preventing it, and in strengthening the bases of our public health system. But without the hearty co-operation of the people generally that system can never accomplish all that it is capable of achieving, and there is no more fruitful method of bringing home to the populace their duty to themselves and their fellows than by the organisation of Health Weeks all over the country. In this matter each of us is his brother's keeper. An enlightened selfishness may sometimes be the purest altruism.

### PEMBREY AND PARADOX.

DR. PEMBREY, the distinguished Professor of Physiology in the University of London, has been making the flesh of Sheffield creep and enjoying himself amazingly in the process. He has been telling the Rotary Club of that city various things they did not know, and are surprised to learn. Milkmen with dirty finger nails are to be encouraged, because clean nails would be a proof of laziness. The average woman prefers a motor-car to a baby and a perambulator. It is nonsense to suppose that you get better babies by feeding expectant mothers. Cottages should be sanitary, but there is no necessity for them to be watertight. There is no co-education in the Medical School at Guy's—his students "have quite enough distractions with the nurses, without women students." Woman is superior to man—which is "what every woman knows." These little paradoxes occurred in an address on "The Application of Biology to Social

Problems." We should be sorry to criticise any of them, since it would be at once unwise and ungracious to do anything calculated to dry up the stream of paradox when it flows so copiously. We do not, however, quite follow Professor Pembrey when he declares that when the milkman has clean nails the price of milk goes up; but that is just the beauty of a paradox—you cannot follow it. Its elusiveness and the new light it throws upon accepted facts are highly stimulating to the mind. The man, for instance, who should turn a familiar copybook heading into "Punctuality is the thief of time" would be not only an "ingenious chiel" himself, but the cause of a useful train of philosophic speculation in others.

### SURGEON AND MAN OF LETTERS.

FROM Sir Thomas Browne downwards there have been many doctors who have written well, but the number of men who have been at once brilliant operators and brilliant men of letters is necessarily small, since surgery as an art is much more modern than writing as an art. The late Sir Frederick Treves was the outstanding example of one who, although essentially a man of letters, achieved his principal fame as a surgeon. So great, indeed, was that fame that even one of so firm a mind must have found it difficult to retire in his prime because he was convinced that no man ought to operate after five-and-fifty. This retirement gave him leisure to write books, some of which will take long to lose their charm. "The Story of the Elephant Man," which appeared little more than six months ago, while it told of a grim human tragedy, revealed at the same time how crude were the hospital and surgical methods of much less than half a century ago.

### THE REFORMED PUBLIC-HOUSE.

THE correspondence on this subject which we have been printing recently illustrates some of the difficulties in the way of placing the sale of drink on a more satisfactory footing. The cause of Temperance has been sadly hampered by the insistance of misguided enthusiasts upon a degree of control for which the country is not, and probably never will be, prepared. To many people "Temperance" means total abstinence, which is an abuse of the English language as well as an itch for the unattainable. All this is the greater pity since real temperance is so closely bound up with the health of the people. We are a much more abstemious nation than we were in the last century, or even at the beginning of the present; but the disease, poverty, and misery caused by drunkenness are still of vast volume. Undue regulation, or anything in the shape of local option, as we have just seen in Scotland, does not grow in favour, while there is little to be said for the creation of a State monopoly. The most certain and the easiest method of keeping drink in its proper place is the wide adoption of the reformed public-house. The existence of all manner of divisions—"Snuggeries," "Lounges," "Bar Parlours" and the rest—is a distinct incentive to furtive drinking. All these things need to be swept away and to be replaced by something more open and more in conformity with the better public conscience. The various Public-house Trusts have shown that this can be done with pecuniary success. The hasty gulping down of glasses of beer or spirits is an insane and deleterious practice; but a glass of sound beer, slowly consumed over a newspaper or a contemplative or observant pipe, is unlikely to do any harm to anybody.

### FOOT-AND-MOUTH DISEASE.

AT the annual dinner of the Royal Society of Medicine, the President, Sir William Hale-White, made the interesting statement that the Society's Section of Comparative Medicine is especially devoting itself to the study of diverses which are, or may be, common to men and animals. The Minister of Health, who was one of the guests, improved the occasion to ask the help of the doctors to find a remedy for, or a means of controlling, foot-and-mouth disease. A fresh and virulent epidemic of that highly infectious disease has been raging for some time, and is costing the country nearly half-a-million a week in compensation for slaughtered cattle and sheep. The policy of slaughter has been much criticised, but the Ministries of Health and of Agriculture and the Royal Agricultural Society are all strongly in favour of its continuance. The alternative is isolation, which has never succeeded save on a small scale, as in the case of a pedigree herd. The countries that have tried it have spent large sums, with the result that in most cases the disease has become endemic. These epidemics are a serious threat to the supply both of meat and milk, and they also threaten, happily in small degree, to extend to man. The Minister of Health has issued a statement showing that, although foot-and-mouth disease can be communicated by animals to man, mainly through the secretions from the mouth of the infected beasts to those in close contact with them, such cases are rare and mild. Slaughter is obviously a precaution against the spread of the disease to human kind. Now that the help of experimental medicine has been formally invoked, we may hope that some real progress will speedily be made towards the control of an insidious and costly plague.

### FOG AS "POISON GAS."

WHAT the recent fogs mean to London is made startlingly clear by the official figures of the death-rate. Between November 17 and December 1 the general death-rate of the capital increased by practically 50 per cent.; the deaths from bronchitis and broncho-pneumonia went up from 106 to 251, and those from heart disease from 186 to 272. In the week ending December 8, the deaths from affections of the lungs were even higher, but those from heart disease fell a little. We cannot be sure that there will be no return of fog this winter; experience, in fact, suggests that the return is unlikely to be confined to the singular number. It is true that London does not stand alone in its liability to fogs; they afflict, more or less seriously, the whole of the Thames Valley as well as the great industrial towns; but when fog is mixed with stagnant smoke the consequences to health must inevitably be serious. It is only by reducing smoke that we can reduce fog from a danger to an inconvenience, and that is by no means an easy matter. There is a good deal of loose talk about "abolishing the open fire," but that is not a practicable proposal. If factory chimneys can consume their own smoke, or render it innocuous, it is to be supposed that household chimneys can be made to do likewise. This is a more practical approach to the solution of the difficulty than the constant urging of the general adoption of some form of radiator. Between being suffocated by hot air indoors and poisoned by smoke-laden fog outside is a hard alternative. Nor need it be a necessary alternative. It is surely within the possibilities of science to do for us something better than either.

### A HOME FOR EVERY CHILD.

"EVERY necessitous widow should be paid to look after her own children at home in the country if possible," said Dr. Scurfield, late Medical Officer of Health of Sheffield, in the course of an interesting lecture delivered in that city in connection with the winter's course on Health Education. He went on to suggest that if a widow had only one child she could easily be employed as a foster-mother for other motherless children, as is done frequently in the United States. The Child Welfare Movement is still comparatively in its infancy, and though we are sensible of the bad effects upon children of institutional life in towns, which gives the mind as little chance of healthy expansion as the body, the possibilities of the boarding-out system have never been fully explored. As Dr. Scurfield points out, we have not paid enough to get the right kind of mother and home, and yet without these the system is worse than useless. In this connection he mentions the colony at Horsham, one of those established by the United Services Guild, where the foster-mother is paid 15s. per week for the first child, 14s. for the second, and 13s. for the third. The accommodation is excellent, the children are happy and healthy. Dr. Scurfield suggests that the Child Welfare Committee of each industrial town might have a ring of village colonies within, say, a radius of thirty miles, with a resident Health Visitor in each, preferably a Child Welfare worker for her own district. His proposal seems worthy of sympathetic consideration, since only in the life of the home, with its combination of freedom and natural discipline, as apart from artificial rules, can a child grow to healthy maturity.

### ON APPOINTING A PROFESSOR.

THE recent appointment of a new Professor of Medicine in Copenhagen has drawn attention to the strenuous character of the tests to which candidates for Professorships are put in the countries of the extreme north of Europe. There were five Danish doctors willing to succeed Professor Gram, whose name has been so long associated with a certain stain, but only four of them took part in the final heat. A jury of eight distinguished experts sat in judgment for more than a week on these four, who had to give trial or sample lectures, not only on subjects chosen by themselves, but on others chosen for them. The records of the scientific and literary productions of each of the applicants were also scrutinised by this jury, who have recently given their verdict in a majority and a minority report. The majority report is in favour of the appointment of Dr. C. Lundsgaard, while the signatories to the minority report, one of whom is Professor Gram, are not content with expressing their preference for another candidate, Dr. C. Sonne, but insist that one of the trial lectures given by Dr. Lundsgaard was unfortunate with regard to its form as well as its matter. It is evident that a fierce light of publicity is thrown on candidates for professorships in Denmark, and however fair this publicity and the competitive system may be, it is not to be wondered at if some men, well qualified both for research and teaching, shrink from the ordeal. To have to "satisfy the examiners" is a nerve-racking task even for the robust and callow medical student, and after middle age few of us retain that elasticity which buoyed us up when we faced dyspeptic and irritable examiners.

### THE EXTINCTION OF LEPROSY.

LEPROSY is so ancient a disease and, until recently, it has been so intractable that those who have not closely followed the progress that has been made of late years in its treatment are probably startled and incredulous when they are told that there is hope of extinguishing it in the course of the next generation. Yet that, roughly speaking, is the belief of the British Empire Leprosy Relief Association which intends, in the latter part of January, to call prominent attention, by means of a Mansion House meeting and a broadcasted appeal, to what it is doing. Now that, thanks to Sir Leonard Rogers and other students and experimenters, it is clear that leprosy is curable, it has set before it the noble aim of stamping it out in the Empire. There are a few—a very few—lepers in England, but there are more than 300,000 in the Empire, most of whom cannot, at present, be reached by curative means, notwithstanding all that is being done by a great many agencies of beneficence. Segregation is an essential for ridding the world of this terrible disease, but neither segregation nor medicinal treatment on a large scale is possible without money. The Association is asking for "at least" a quarter of a million with which to supplement Government, charitable and religious work. That is just about a pound a leper, and, when it is put in that way, the duty of providing these pounds ought to come home to great numbers of people. Britain has set a good example in its dealings with these unhappy people, the outcasts of history, and the more rapid and complete our own success the more likely other countries are to follow in our footsteps.

### THE FATAL GEYSER.

BATH-ROOM tragedies, caused by inhaling the fumes from geysers, appear to be increasing in frequency. It is almost inevitable that they should increase. Not only are most geysers fixed without a flue to carry away the products of combustion, but this method of heating water is growing in popularity. At one time it was a makeshift, adopted because it was costly or impracticable to lay hot-water pipes from the kitchen range to the bathroom. Now, however, that the gas-cooker enjoys such signal favour, and that the wasteful circulating boiler is looked at askance, geysers are being put into the bath-rooms of many new houses. It should be clearly understood that no geyser is safe without a flue. An open window will, no doubt, serve the purpose of ventilation, but bath-room windows may remain closed owing to the carelessness or forgetfulness of the bather, nor is it always agreeable to take a bath on a cold winter morning with the window open. There is no difficulty whatever about baffling down-draughts in the flue or preventing the accumulation of soot, and, with a little care, absclute safety can be assured. Possibly some official action may yet be called for, but in the meantime gas companies might surely refuse to connect supplies unless efficient ventilating arrangements were installed. In the latest tragedy we have seen reported, a husband who went to rescue his wife from the deadly fumes, was himself overcome, and both were suffocated.

### IS TWILIGHT SLEEP WANING?

GERMANY is the home of twilight sleep, and it is interesting to note that the most recent reports from Freiburg and other centres in Germany are not as enthusiastic as they were a few years ago. It would seem that many women, whose confinements had been conducted under twilight sleep, have flatly refused to submit to it again, preferring the pain of the known to the nebulous memories of the unknown. The term " twilight sleep " is apt to be a misnomer, for some accouchers complain that the patient is often so violent while in this half-conscious state that it is difficult or impossible to carry out certain supplementary helpful measures. In Germany, as in other countries, hypnosis has also lately been given an extensive trial in labour, and when the desired effects have not been achieved, the patients have been assured that failure to banish pain depended on their own contumaciousness and hysteria. Considering that this treatment necessitates a preparatory training of the patient for about a fortnight, the general adoption of hypnosis would seem to be impracticable even if it were in other respects desirable. But this does not seem to be the case. Hypnosis in connection with child-birth has recently been defined in Germany as a psychic trauma and, as in the case of twilight sleep, the mothers themselves have signified their disapproval. In view of these experiences in Germany, it must be confessed that the problem of painless labour has not yet been solved to the satisfaction of all concerned.

### THE PRINCE OF WALES AND SIR BENJAMIN BRODIE.

THE Prince of Wales is a very ingenuous young man—that is one reason why he is so engaging. If he does not know a thing he says so quite artlessly, yet it is a little startling to find him telling the Royal Society of Medicine at its annual dinner: " I don't know much about Sir Benjamin Brodie, but I feel sure he was a very wise old man." The famous surgeon had, it seems, advised young men to abstain from medical politics and to keep clear of " exciting and irritating discussions." There is no doubt about Sir Benjamin's wisdom, but there are so many exciting, if not irritating, discussions in medical politics in these days that we doubt whether even this wise old man would have been able to avoid them. It is pleasant to know that there is still a Sir Benjamin Brodie, the third of the name, and curious to observe the odd circumstance that this distinguished scientific family—for the second baronet was Waynflete Professor of Chemistry in the University of Oxford—still retains its affinities with the word " Serjeant." The " wise old man " was Serjeant-Surgeon to William IV. and Queen Victoria, and he married the daughter of a Serjeant-at-Law. His eldest son also married the daughter of a Serjeant-at-Law, and his grandson, the third baronet, married the daughter of the Serjeant-at-Arms to Queen Victoria. The old writers on tenures would have called this Grand Serjeantry with a vengeance.

### DEATHS FROM POISONING.

WE are so apt to jump to conclusions as to the facts and the drift of the everyday life about us that the Registrar-General's Report, when it appears, is invariably a useful corrective. In that recently published it is curious to note that, in spite of the great increase of female labour, and the entry of women generally into more strenuous spheres, the death-rate among women is still about 15 per cent. lower than among men. Poisoning, as a cause of death, shows a definite increase, but it is surprising that in more than one-quarter of the cases of taking poison was accidental; there is indeed need for precautions. The greatest number of fatalities occurred from hydrochloric and carbolic acids, and in view of these figures we are certainly tempted to suggest, even though these substances are useful for many essential purposes, that the regulations governing their distribution and sale require some measure of revision.

### CAN SYPHILIS BE STAMPED OUT?

PROBABLY this would seem to be the logical inference to be drawn from a paper recently read by Professor C. Rasch at one of the celebrations in France of the Pasteur centenary. His account of the venereal disease campaign in Denmark showed that, as in every other campaign, money—much money—is needed. But whereas many campaigns give no monetary return, that in Denmark against venereal disease is already adding to the wealth of the nation by an appreciable decrease of sickness due to syphilis and allied diseases. At present the anti-venereal disease campaign costs about half-a-crown in English money per head of population. It is noteworthy that in 1921 there were 1,942 new cases of syphilis, whereas in 1922 the number had fallen to 1,578. At this rate of decline it is easy to calculate that syphilis will cease to count as a national scourge during the life of the present generation. Many factors have contributed to the decline in its frequency during the last few years, and probably the most important factor is the wholesale use of the salvarsan group of drugs. But as the incidence of gonorrhœa is also declining, and as it is not influenced by salvarsan, it is probable that the falling incidence of venereal disease is to a considerable extent due to other factors, such as the increasing use of devices for the prevention of infection.

